# Association Between Maternal Perceived Stress in All Trimesters of Pregnancy and Infant Atopic Dermatitis: A Prospective Birth Cohort Study

**DOI:** 10.3389/fped.2020.526994

**Published:** 2020-11-16

**Authors:** Qianwen Shen, Qianqian Zhang, Jiuru Zhao, Zhen Huang, Xiaoli Wang, Meng Ni, Zheng Tang, Zhiwei Liu

**Affiliations:** ^1^Department of Neonatology, International Peace Maternity and Child Health Hospital, Shanghai Jiao Tong University School of Medicine, Shanghai, China; ^2^Shanghai Key Laboratory of Embryo Original Disease, International Peace Maternity and Child Health Hospital of China Welfare Institute, Shanghai, China; ^3^Shanghai Municipal Key Clinical Specialty, Shanghai, China

**Keywords:** maternal perceived stress, trimesters, offspring, atopic dermatitis, birth cohort

## Abstract

**Background:** Currently, most studies indicate that there is a potential link between maternal psychologic stress and the risk of atopic dermatitis (AD) in offspring. However, it is unknown which trimester of pregnancy is most sensitive to maternal stress in terms of risk of infant AD and whether the changes of maternal stress level in different trimesters of pregnancy may be associated with infant AD. In this study, we aimed to investigate the association between maternal perceived stress across three trimesters of pregnancy and AD in infants at 6 months.

**Methods:** A total of 1,638 pregnant women participated in the population-based birth cohort study. Maternal prenatal stress was assessed by self-report questionnaires during each trimester. Infant AD was diagnosed at age 6 months, according to the UK Working Party diagnostic criteria. Univariate and multivariate logistic regression models were used to analyze the association between maternal prenatal stress in each trimester of pregnancy and infant AD.

**Results:** Maternal perceived stress in the 2nd trimester was associated with AD in infants at 6 months (aOR 1.56; 95% CI 1.08–2.25, *P* = 0.019). Furthermore, increased level of perceived stress from the 1st to the 2nd trimester (aOR 2.05, 95% CI 1.33–3.15, *P* = 0.001) and from the 1st to the 3rd trimester (aOR 1.92, 95% CI 1.22–3.00, *P* = 0.004) were also associated with the risk of infant AD at 6 months.

**Conclusion:** A high level of maternal perceived stress in the 2nd trimester and increased level of perceived stress from the 1st to the 2nd and 3rd trimesters of pregnancy may increase the risk of offspring developing AD at 6 months.

## Introduction

Atopic dermatitis (AD), also known as eczema, characterized by itchy rash, flexural rash, and generally dry skin, has become one of the most common chronic disease in childhood (1). Its prevalence has increased rapidly in many countries over the past decades (2, 3), leading to a decreased quality of life for children and an increased financial burden for their families (4). In developed countries, more than 20% of children are affected by AD (5). In China, its prevalence is 12% among urban preschool children (6). Moreover, AD is often an early sign of atopic march toward allergic diseases, such as allergic rhinitis and asthma (7, 8). It is now believed that AD is a result of genetic, environmental, and immunological factors (9). Therefore, it is important to identify the factors that are associated with childhood AD, as addressing those factors may help prevent the disease.

Recently, an increasing number of studies have focused on the effects of the early intrauterine environment on offspring. Most studies have reported that maternal prenatal psychologic stress is associated with atopic disease in the offspring. For example, one study showed that children whose mothers had experienced stressful life events during pregnancy had a moderately increased risk of having wheezing, asthma, AD, and allergic rhinitis during childhood (10). There is also growing evidence of a potential link between maternal psychologic stress and the risk of AD in offspring (11–14).

However, most studies evaluated maternal prenatal stress at only one stage during pregnancy. Few studies have continuously evaluated stress throughout pregnancy and assessed the effects of changes in maternal stress on the infants' risks of AD (15, 16). Pregnancy is a dynamic process, and maternal prenatal stress constantly changes (17). Stress during different trimesters has varying effects on fetal development. For example, stress in early pregnancy often leads to pregnancy loss, while stress in late pregnancy may lead to low birth weight, which is a risk factor for diseases in adulthood (18). At present, it remains unknown which trimester of pregnancy is most sensitive to maternal stress and the subsequent risk of infant AD. It is also unclear whether changes in maternal stress during pregnancy are associated with infant AD. Therefore, this study aimed to investigate the association between maternal prenatal stress during the whole pregnancy and the risk of AD in the infants.

## Materials and Methods

### Study Design

This study was conducted as part of the China National Birth Cohort study. Ethics approval was obtained from the Ethics Committees of the International Peace Maternity and Child Health Hospital (IPMCH) affiliated with Shanghai Jiao Tong University School of Medicine [approval number (GKLW) 2016-21]. All subjects provided written informed consent in accordance with the Declaration of Helsinki.

Women were recruited after a confirmed pregnancy at IPMCH from October 2017 to March 2019. The inclusion criteria were: (1) long-term resident of Shanghai; (2) ability to read and write Chinese; (3) no history of mental illness or severe mental trauma; and (4) antenatal care and delivery at IPMCH. The exclusion criteria were: (1) stillborn or multiple fetuses and (2) the time of delivery was earlier than the third follow-up.

Basic information such as age, ethnicity, education, parity, gestational disease, and health status were recorded by trained personnel at the first follow-up visit after registration in the obstetrics department in the early period of pregnancy. A standardized questionnaire was used to evaluate perceived stress (described below). Follow-up was continued during the second (24–26 weeks) and third trimesters (30–32 weeks), using the same questionnaire to assess stress. All pregnancy follow-ups were conducted when the women came to the hospital for antenatal examination and the questionnaires were self-administered. After birth, data on each newborn, such as sex, date of birth, birth weight, delivery mode, and gestational age were collected from the hospital medical record. At 6 months postpartum, mothers and their babies returned to the hospital to conduct a follow-up medical examination for the child. Children's health data including body length, weight, head circumference, feeding style, history of probiotics supplement and antibiotic use, and assessment of AD in the past 6 months were recorded by parent-completed questionnaire.

**Figure 1:**
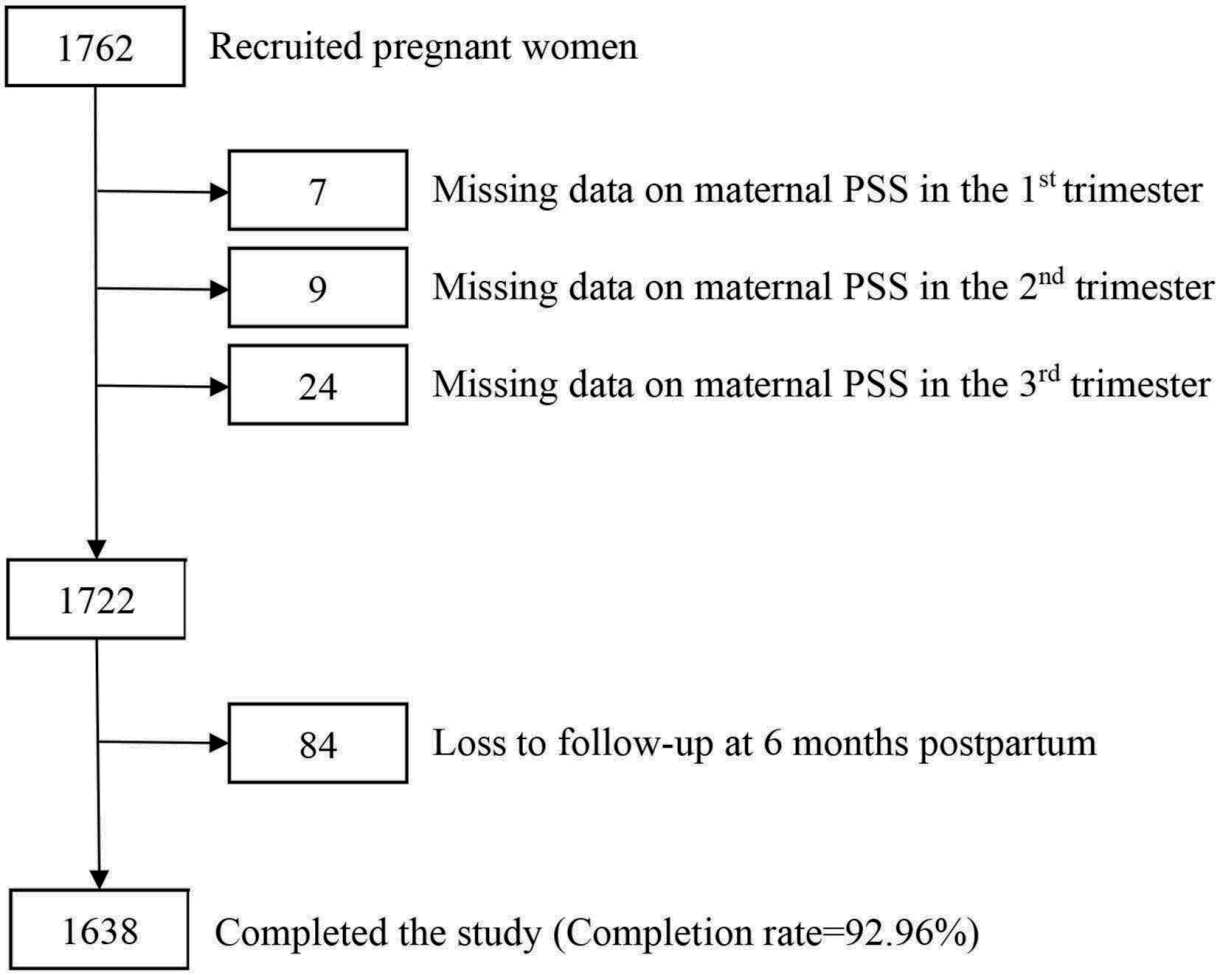
Flow chart of participants in the study.

For the current study, 1,762 pregnant women who delivered at IPMCH were recruited and followed up until 6 months postpartum. Finally, 1,638 pregnant women completed the study. The study completion rate was 92.96%. The flow chart of participants are shown in Figure 1.

### Assessment of Maternal Prenatal Stress

Maternal prenatal stress was assessed during each trimester (between the 8th and 10th week, 24th and 26th week, and 30th and 32nd week of gestation, for the 1st, 2nd, and 3rd trimesters, respectively) by self-report questionnaires using the 10-item Perceived Stress Scale (PSS) (19). PSS, which has been widely used worldwide, assesses the extent to which respondents think their lives have been unpredictable, uncontrollable, and overloaded in the previous month and evaluates the extent to which external demands seem to exceed the individual's perceived ability to cope. This scale uses a Likert-type five-point scale (never, almost never, sometimes, fairly often, very often) and yields a total score range of 0–40. A higher score indicates a higher degree of perceived stress. Scale reliability is considered acceptable when Cronbach's alpha ≥ 0.70 (20). The reliability of PSS has been verified among pregnant women at risk for mental distress (Cronbach's alpha = 0.88) (21). In this study, we used the median score from each trimester to categorize PSS scores into high and low levels and the alteration in PSS level in different trimesters to evaluate changes in maternal prenatal stress during pregnancy.

### Diagnosis of AD

AD was assessed at 6 months, based on the UK Working Party diagnostic criteria (22), using a standardized questionnaire. Children were classified as having AD at 6 months if parents affirmed a history of itchy skin condition in addition to two of the following: a history of flexural involvement, a history of a generalized dry skin, and visible flexural dermatitis.

### Statistical Analysis

Student's *T*-tests were used to compare continuous variables, and Chi-square tests and Fisher's exact probability tests were used for categorical variables. The outcome of AD was analyzed as a binary variable. Odds ratios (ORs) and 95% confidence intervals (CIs) were calculated for the maternal prenatal perceived stress and infants AD in each trimester of pregnancy using logistic regression models with and without adjustment for confounders. A *P* < 0.05 was considered statistically significant. Subjects with missing data about infant AD and/or maternal prenatal stress were excluded from the analysis.

In the logistic regression analysis, potential confounding factors, which may be associated with maternal stress and infant AD, included maternal age at delivery, ethnicity, education, family income, parity, gestational diabetes mellitus, gestational hypertension, and parental history of allergic diseases (23, 24). Using the daggity.net program, a directed acyclic graph (DAG) was built to describe the relation between study variables for maternal prenatal stress and infant AD (the diagram is shown in Supplementary Figure 1).

In addition, we tried to add some potential confounding factors, which were selected according to the reference (25), such as infant sex, delivery method, birth season, feeding pattern, and use of probiotics and antibiotics, to adjust in another logistic regression analysis.

Statistical analyses were performed using SPSS 24.0 (IBM Corp., Armonk, NY, USA).

## Results

In this study, 1,762 neonates were recruited, of which 1,638 were included in the final analysis. There was no difference in characteristics between the recruited and study populations (the result was shown in Supplementary Table 1).

The characteristics of mothers and infants are shown in Table 1. A total of 134 (8.2%) infants were diagnosed with AD at 6 months. The effects of potential confounding variables for infant AD are presented. Parental history of allergic diseases, birth season, and infant sex were associated with the risk of AD (*P* < 0.05).

**Table 1:** Characteristics of the study population^a^.

**Characteristics**	***N* = 1,638**	**With AD at 6 months, *N* = 134**	**Without AD at 6 months, *N* = 1,504**	***P*-value**
Maternal age at delivery (years)	31.6 (4.1)	32.0 (4.2)	31.6 (4.1)	0.273
Maternal ethnicity				0.090
Han nationality	1,610 (98.3)	134 (100.0)	1,476 (98.1)	
The minority	28 (1.7)	0 (0.0)	28 (1.9)	
Maternal education				0.135
Master's degree or higher	351 (21.4)	23 (17.2)	328 (21.8)	
College degree	1,180 (72.0)	106 (79.1)	1,074 (71.4)	
Less than college	107 (6.6)	5 (3.7)	102 (6.8)	
Family income (CNY yuan)				0.893
0–100,000	118 (7.2)	11 (8.2)	107 (7.1)	
100,000–300,000	1,009 (61.6)	82 (61.2)	927 (61.6)	
>300,000	511 (31.2)	41 (30.6)	470 (31.3)	
Gestational diabetes mellitus				0.186
Yes	220 (13.4)	23 (17.2)	197 (13.1)	
No	1,418 (86.6)	111 (82.8)	1,307 (86.9)	
Gestational hypertension				0.250
Yes	22 (1.3)	0 (0.0)	22 (1.5)	
No	1,616 (98.7)	134 (100.0)	1,482 (98.5)	
Parity				0.757
Multiparous	496 (30.3)	39 (29.1)	457 (30.4)	
Primiparous	1,142 (69.7)	95 (70.9)	1,047 (69.6)	
Parental history of allergic diseases				** <0.001**
Yes	460 (28.1)	83 (61.9)	377 (25.1)	
No	1,178 (71.9)	51 (38.1)	1,127 (74.9)	
Infant sex				**0.049**
Male	832 (50.8)	79 (59.0)	753 (50.1)	
Female	806 (49.2)	55 (41.0)	751 (49.9)	
Birth weight (grams)	3331.1 (429.7)	3381.0 (428.2)	3326.6 (429.6)	0.160
Gestational age (weeks)	38.9 (1.3)	39.0 (1.2)	38.9 (1.3)	0.537
Delivery method				0.244
Cesarean section	714 (43.6)	52 (38.8)	662 (44.0)	
Vaginal delivery	924 (56.4)	82 (61.2)	843 (56.0)	
Birth season				**0.001**
Spring	288 (17.6)	12 (9.0)	276 (18.4)	
Summer	288 (17.6)	17 (12.7)	271 (18.0)	
Autumn	443 (27.0)	52 (38.8)	391 (26.0)	
Winter	619 (37.8)	53 (39.5)	566 (37.6)	
Feeding pattern				0.822
Formula feeding	275 (16.8)	24 (17.9)	251 (16.7)	
Mixed feeding	551 (33.6)	47 (35.1)	504 (33.5)	
Breast feeding	812 (49.6)	63 (47.0)	749 (49.8)	
Use of probiotics regularly during 6 months				0.784
Yes	145 (8.9)	11 (8.2)	134 (8.9)	
No	1,493 (91.1)	123 (91.8)	1,370 (91.1)	
Use of antibiotics during 6 months				0.074
Yes	130 (7.9)	16 (11.9)	114 (7.6)	
No	1,508 (92.1)	118 (88.1)	1,390 (92.4)	

a *Categorical variables are shown as numbers (percentage) and continuous variables are shown as mean (standard deviation)*.

*AD, atopic dermatitis. Bold values mean P < 0.05*.

The results of the univariate (unadjusted) and multivariate (adjusted) analyses of the relationship between maternal prenatal perceived stress level across trimesters and infant AD at 6 months are shown in Table 2. Infant AD at 6 months was associated with maternal perceived stress in the 2nd trimester (OR 1.48, 95% CI 1.04–2.12, *P* = 0.029). After adjusting for potential confounders, the risk of AD remained associated with maternal perceived stress in the 2nd trimester (OR 1.56, 95% CI 1.08–2.25, *P* = 0.019), but not in the 3rd trimester (OR 1.23, 95% CI 0.86–1.78, *P* = 0.261). Furthermore, this association did not exist in infants with a higher level of maternal perceived stress in the 1st trimester, in either the univariate (OR 0.73, 95% CI 0.51–1.05, *P* = 0.089) or multivariate (OR 0.73, 95% CI 0.50–1.07, *P* = 0.106) analyses.

**Table 2:** Relationship between maternal prenatal perceived stress level across the trimesters of pregnancy and infant AD at 6 months of age.

**Maternal prenatal perceived stress level**	**With AD at 6 months, N (%)**	**Unadjusted, OR (95% CI)**	***P-*value**	**Adjusted^b^, OR (95% CI)**	***P*-value**
In the 1st trimester			0.089		0.106
Low	83 (9.2)	Reference		Reference	
High	51 (6.9)	0.73 (0.51–1.05)		0.73 (0.50–1.07)	
In the 2nd trimester			**0.029**		**0.019**
Low	59 (6.8)	Reference		Reference	
High	75 (9.8)	1.48 (1.04–2.12)		1.56 (1.08–2.25)	
In the 3rd trimester			0.255		0.261
Low	68 (7.5)	Reference		Reference	
High	66 (9.0)	1.23 (0.86–1.75)		1.23 (0.86–1.78)	

b *Adjusted for maternal age at delivery, ethnicity, education, family income, parity, gestational diabetes mellitus, gestational hypertension, and parental history of allergic diseases*.

*AD, atopic dermatitis; OR, odds ratio; CI, confidence interval. Bold values mean P < 0.05*.

Similar analyses of the relationship between the changes in maternal stress level during different trimesters and infant AD at 6 months are shown in Table 3. Increased perceived stress from the 1st to the 2nd trimester was associated with a higher risk of infant AD at 6 months (OR 2.06, 95% CI 1.37–3.11, *P* < 0.001). Moreover, increased perceived stress from the 1st to the 3rd trimester also showed an association with a higher risk of infant AD at 6 months of age (OR 1.82, 95% CI 1.19–2.80, *P* = 0.005). Both associations remained significant after adjusting for confounding variables (OR 2.05, 95% CI 1.33–3.15, *P* = 0.001 and OR 1.92, 95% CI 1.22–3.00, *P* = 0.004). However, there was no association between increased maternal perceived stress from the 2nd to the 3rd trimester in both the univariate (OR 1.02, 95% CI 0.59–1.77, *P* = 0.935) and multivariate analyses (OR 0.99, 95% CI 0.56–1.74, *P* = 0.967).

**Table 3:** Relationship between alterations in maternal PSS level during different trimesters and infant AD at 6 months.

**Alterations of maternal perceived stress level**	**With AD at 6 months, N (%)**	**Unadjusted, OR, 95%CI**	***P-*value**	**Adjusted^b^, OR, 95%CI**	***P-*value**
From the 1st to the 2nd trimester			** < 0.001**		**0.001**
Not increased	99 (7.2)	Reference		Reference	
Increased	35 (13.7)	2.06 (1.37–3.11)		2.05 (1.33–3.15)	
From the 2nd to the 3rd trimester			0.935		0.967
Not increased	118 (8.2)	Reference		Reference	
Increased	16 (8.3)	1.02 (0.59–1.77)		0.99 (0.56–1.74)	
From the 1st to the 3rd trimester			**0.005**		**0.004**
Not increased	103 (7.4)	Reference		Reference	
Increased	31 (12.7)	1.82(1.19–2.80)		1.92 (1.22–3.00)	

b *Adjusted for maternal age at delivery, ethnicity, education, family income, parity, gestational diabetes mellitus, gestational hypertension, and parental history of allergic diseases*.

*AD, atopic dermatitis; OR, odds ratio; CI, confidence interval; PSS, perceived stress scale. Bold values mean P < 0.05*.

In addition, the result which used added confounding factors was similar (the result is shown in Supplementary Tables 2, 3).

## Discussion

In this birth cohort study, we found that maternal perceived stress in the 2nd trimester of pregnancy, but not during the 1st and 3rd trimesters, was associated with a risk of AD in the offspring. Interestingly, we also found that increased maternal perceived stress from the 1st to the 2nd and 3rd trimesters was associated with a higher risk of infant AD at 6 months. However, there was no association between increased maternal perceived stressed from the 2nd to the 3rd trimester of pregnancy and risk of infant AD at 6 months.

We found that maternal perceived stress only in the 2nd trimester of pregnancy was associated with a risk of infant AD. Similarly, previous research has reported that the risk of AD at the age of 14 years increased with an elevated number of adverse life events experienced by the mothers between gestational weeks 18 and 34, but not between conception and gestational weeks 18 (26). The mechanism underlying the influence of maternal prenatal stress on infant risk of AD during a specific period is still unclear. It has been suggested that there are windows of vulnerability during immune system development (27). Coussons-Read et al. (28) reported that a high level of stress was related to higher serum interleukin (IL)-6 levels in the 1st and 3rd trimesters, and lower IL-10 levels in the 1st trimester. Additionally, high stress in the 2nd trimester was associated with increased serum levels of C-reactive protein. These inflammatory response alterations during pregnancy may affect the developing immune system of the fetus and lead to susceptibility to atopic diseases in offspring. AD, an inflammatory disease, is largely due to an inappropriate immune system response.

In addition, we found that the risk of infant AD is associated with increased maternal stress from the 1st to the 2nd and 3rd trimesters, but not with increased stress from the 2nd to 3rd trimester. The potential mechanisms for this may involve neuroendocrine responses. Maternal psychologic stress can activate the central nervous system and hypothalamic-pituitary-adrenal (HPA) axis, leading to increased release of corticosteroids. Excessive corticosteroid concentrations can disrupt the fetal HPA axis, resulting in continual activation of the fetus' HPA axis (29). Increased glucocorticoids may regulate IL-4, IL-10, and IL-13 production, skewing the system toward attenuation of T helper 1-dependent responses and production of T helper 2 responses, which may lead to allergic inflammation (30). Stress during early pregnancy has been reported to cause robust activation of the maternal HPA axis; however, maternal HPA axis responses to stress is remarkably attenuated after mid-pregnancy (31). Nevertheless, the effects of the changes in maternal stress on the HPA axis need to be further clarified.

The major advantage of this study is using the same tool to measure maternal perceived stress at various times during pregnancy. In addition, we used data from a population-based birth cohort study with detailed information on the maternal psychosocial characteristics and offspring's characteristics. However, there are some limitations. First, in this study, maternal perceived stress and infant AD were based on self-reported information; thus, recall bias might exist. The possible reporting bias may bring non-differential misclassification, which could bias the effect estimate toward none. Second, the follow-up was only until 6 months of age, and some children may have been diagnosed with AD after the age of 6 months. Therefore, the long-term effect of increased maternal stress across pregnancy on the risk of AD in childhood could not be determined. Furthermore, no measures were made for postnatal maternal stress. Future studies should take this factor into account. In addition, although we have tried our best to minimize the chance of the selection bias in the recruitment of the study subjects, the selection bias may still exist.

In conclusion, we found that high levels of maternal perceived stress in the 2nd trimester of pregnancy was a risk factor for infant AD development. Moreover, increased perceived stress from the 1st to subsequent trimesters may play an important role in infant AD. This study may provide potential strategies to reduce the risk of infant AD through the maternal perceived stress factors. Future studies are needed to evaluate the association of maternal psychologic stress in different periods of pregnancy and the development of allergic diseases in offspring.

## Data Availability Statement

The datasets generated for this study are available on request to the corresponding author.

## Ethics Statement

The studies involving human participants were reviewed and approved by Ethics Committees of the International Peace Maternity and Child Health Hospital affiliated with Shanghai Jiao Tong University School of Medicine. The patients/participants provided their written informed consent to participate in this study. Written informed consent was obtained from the individual(s) for the publication of any potentially identifiable images or data included in this article.

## Author Contributions

QS conceptualized and designed the study and drafted the initial manuscript. ZH designed the study and completed the statistical analyses. QZ and JZ conceptualized and designed the study and assisted with the statistical analyses. XW and MN designed the study and collected the data. ZT and ZL conceptualized and designed the study, coordinated and supervised data collection, and critically reviewed and revised the manuscript. All authors were involved in revising the paper, approved the submitted version, and agreed to be accountable for all aspects of the work.

## Conflict of Interest

The authors declare that the research was conducted in the absence of any commercial or financial relationships that could be construed as a potential conflict of interest.

## Abbreviations

AD: atopic dermatitis
CI: confidence interval
DAG: directed acyclic graph
HPA: hypothalamic-pituitary-adrenal
IL: interleukin
IPMCH: International Peace Maternity and Child Health Hospital
OR: odds ratio
PSS: perceived stress scale.
