# Supplementary material for: Association Between Maternal Perceived Stress in All Trimesters of Pregnancy and Infant Atopic Dermatitis: A Prospective Birth Cohort Study
Source: Front Pediatr. 2020 Nov 16;8:526994. doi: 10.3389/fped.2020.526994 (PMC7701332; doi:10.3389/fped.2020.526994)
Supplement: Supplementary file 1 [file Table_1.DOCX]

**Table S1. Comparison of the** **recruited population and the study population**

| **Characteristics** | **Recruited (N=1762)** | **Study (N=1638)** | ***P* value** |
| --- | --- | --- | --- |
| Maternal age at delivery (years) | 31.6 (4.1) | 31.6 (4.1) | 0.756 |
| Maternal ethnicity |  |  | 0.720 |
| Han nationality | 1729 (98.1) | 1610 (98.3) |  |
| The minority | 33 (1.9) | 28 (1.7) |  |
| Maternal education |  |  | 0.981 |
| Master’s degree or higher | 376 (21.3) | 351 (21.4) |  |
| College degree | 1268 (72.0) | 1180 (72.0) |  |
| Less than college | 118 (6.7) | 107 (6.6) |  |
| Family income (CNY yuan) |  |  | 0.983 |
| 0-100, 000 | 124 (7.0) | 118 (7.2) |  |
| 100, 000-300, 000 | 1086 (61.7) | 1009 (61.6) |  |
| > 300, 000 | 551 (31.3) | 511 (31.2) |  |
| Gestational diabetes mellitus |  |  | 0.874 |
| Yes | 236 (13.6) | 220 (13.4) |  |
| No | 1497 (86.4) | 1418 (86.6) |  |
| Gestational hypertension |  |  | 0.917 |
| Yes | 24 (1.4) | 22 (1.3) |  |
| no | 1709 (98.6) | 1616 (98.7) |  |
| Parity |  |  | 0.604 |
| Multiparous | 548 (31.1) | 496 (30.3) |  |
| Primiparous | 1214 (68.9) | 1142 (69.7) |  |
| Parental history of allergic diseases |  |  | 0.922 |
| Yes | 467 (27.9) | 460 (28.1) |  |
| No | 1205 (72.1) | 1178 (71.9) |  |
| Infant sex |  |  | 0.740 |
| Male | 905 (51.4) | 832 (50.8) |  |
| Female | 857 (48.6) | 806 (49.2) |  |
| Birth weight (grams) | 3328.8 (434.8) | 3331.1 (429.7) | 0.880 |
| Gestational age (weeks) | 38.9 (1.3) | 38.9 (1.3) | 0.817 |
| Delivery method |  |  | 0.814 |
| Cesarean section | 761 (43.2) | 714 (43.6) |  |
| Vaginal delivery | 1001 (56.8) | 924 (56.4) |  |
| Birth season |  |  | 0.945 |
| Spring | 318 (18.0) | 288 (17.6) |  |
| Summer | 297 (16.9) | 288 (17.6) |  |
| Autumn | 477 (27.1) | 443 (27.0) |  |
| Winter | 670 (38.0) | 619 (37.8) |  |
| Feeding pattern |  |  | 0.978 |
| Formula feeding | 284 (17.0) | 275 (16.8) |  |
| Mixed feeding | 565 (33.8) | 551 (33.6) |  |
| Breast feeding | 823 (49.2) | 812 (49.6) |  |
| Use of probiotics regularly during 6 months |  |  | 0.951 |
| Yes | 147 (8.8) | 145 (8.9) |  |
| No | 1525 (91.2) | 1493 (91.1) |  |
| Use of antibiotics during 6 months |  |  | 0.965 |
| Yes | 132 (7.9) | 130 (7.9) |  |
| No | 1540 (92.1) | 1508 (92.1) |  |
| Maternal prenatal stress level in the 1^st^ trimester |  |  | 0.939 |
| Low | 962 (54.8) | 900 (54.9) |  |
| High | 793 (45.2) | 738 (45.1) |  |
| Maternal prenatal stress level in the 2^nd^ trimester |  |  | 0.693 |
| Low | 915 (52.4) | 869 (53.1) |  |
| High | 832 (47.6) | 769 (46.9) |  |
| Maternal prenatal stress level in the 3^rd^ trimester |  |  | 0.922 |
| Low | 963 (55.6) | 908 (55.4) |  |
| High | 769 (44.4) | 730 (44.6) |  |
| AD at 6 months of age |  |  | 0.939 |
| Yes | 138 (8.3) | 134 (8.2) |  |
| No | 1534 (91.7) | 1504 (91.8) |  |

^a^ Categorical variables are shown as numbers (percentage) and continuous variables are shown as mean (standard deviation)

AD, atopic dermatitis
