# Supplementary material for: Association Between Maternal Perceived Stress in All Trimesters of Pregnancy and Infant Atopic Dermatitis: A Prospective Birth Cohort Study
Source: Front Pediatr. 2020 Nov 16;8:526994. doi: 10.3389/fped.2020.526994 (PMC7701332; doi:10.3389/fped.2020.526994)
Supplement: Supplementary file 2 [file Table_2.DOCX]

**Table S2.** Relationship between maternal prenatal perceived stress level across the trimesters of pregnancy and infant AD at 6 months of age

| **Maternal prenatal perceived stress level** | **With AD at 6 months, N (%)** | **Unadjusted,**  **OR (95% CI)** | ***P* value** | **Adjusted ^b^,**  **OR (95% CI)** | ***P* value** |
| --- | --- | --- | --- | --- | --- |
| In the 1^st^ trimester |  |  | 0.089 |  | 0.071 |
| Low | 83 (9.2) | Reference |  | Reference |  |
| High | 51 (6.9) | 0.73 (0.51−1.05) |  | 0.70 (0.47−1.03) |  |
| In the 2^nd^ trimester |  |  | **0.029** |  | **0.034** |
| Low | 59 (6.8) | Reference |  | Reference |  |
| High | 75 (9.8) | 1.48 (1.04−2.12) |  | 1.51 (1.03−2.20) |  |
| In the 3^rd^ trimester |  |  | 0.255 |  | 0.300 |
| Low | 68 (7.5) | Reference |  | Reference |  |
| High | 66 (9.0) | 1.23 (0.86−1.75) |  | 1.22 (0.84−1.78) |  |

^b^ Adjusted for maternal age at delivery, ethnicity, education, family income, parity, gestational diabetes mellitus, gestational hypertension and parental history of allergic diseases, infant sex, delivery method, birth season, feeding pattern, use of probiotics and antibiotics.

AD, atopic dermatitis; OR, odds ratio; CI, confidence interval.
