# Supplementary material for: Association Between Maternal Perceived Stress in All Trimesters of Pregnancy and Infant Atopic Dermatitis: A Prospective Birth Cohort Study
Source: Front Pediatr. 2020 Nov 16;8:526994. doi: 10.3389/fped.2020.526994 (PMC7701332; doi:10.3389/fped.2020.526994)
Supplement: Supplementary file 3 [file Table_3.docx]

**Supplementary Table 3.** Relationship between alterations in maternal PSS level during different trimesters and infant AD at 6 months

| **Alterations of maternal perceived stress level** | **With AD at 6 months, N (%)** | **Unadjusted,**  **OR, 95%CI** | ***P* value** | **Adjusted ^b^,**  **OR, 95%CI** | ***P* value** |
| --- | --- | --- | --- | --- | --- |
| From the 1^st^ to the 2^nd^ trimester |  |  | **<0.001** |  | **0.001** |
| Not increased | 99 (7.2) | Reference |  | Reference |  |
| Increased | 35 (13.7) | 2.06 (1.37−3.11) |  | 2.09 (1.35−3.25) |  |
| From the 2^nd^ to the 3^rd^ trimester |  |  | 0.935 |  | 0.920 |
| Not increased | 118 (8.2) | Reference |  | Reference |  |
| Increased | 16 (8.3) | 1.02 (0.59−1.77) |  | 1.03 (0.58−1.83) |  |
| From the 1^st^ to the 3^rd^ trimester |  |  | **0.005** |  | **0.003** |
| Not increased | 103 (7.4) | Reference |  | Reference |  |
| Increased | 31 (12.7) | 1.82(1.19−2.80) |  | 2.03 (1.28−3.21) |  |

^b^ Adjusted for maternal age at delivery, ethnicity, education, family income, parity, gestational diabetes mellitus, gestational hypertension and parental history of allergic diseases, infant sex, delivery method, birth season, feeding pattern, use of probiotics and antibiotics.

AD, atopic dermatitis; OR, odds ratio; CI, confidence interval; PSS, perceived stress scale.
