# Supplementary figures and images for: Association Between Maternal Perceived Stress in All Trimesters of Pregnancy and Infant Atopic Dermatitis: A Prospective Birth Cohort Study
Source: Front Pediatr. 2020 Nov 16;8:526994. doi: 10.3389/fped.2020.526994 (PMC7701332; doi:10.3389/fped.2020.526994)

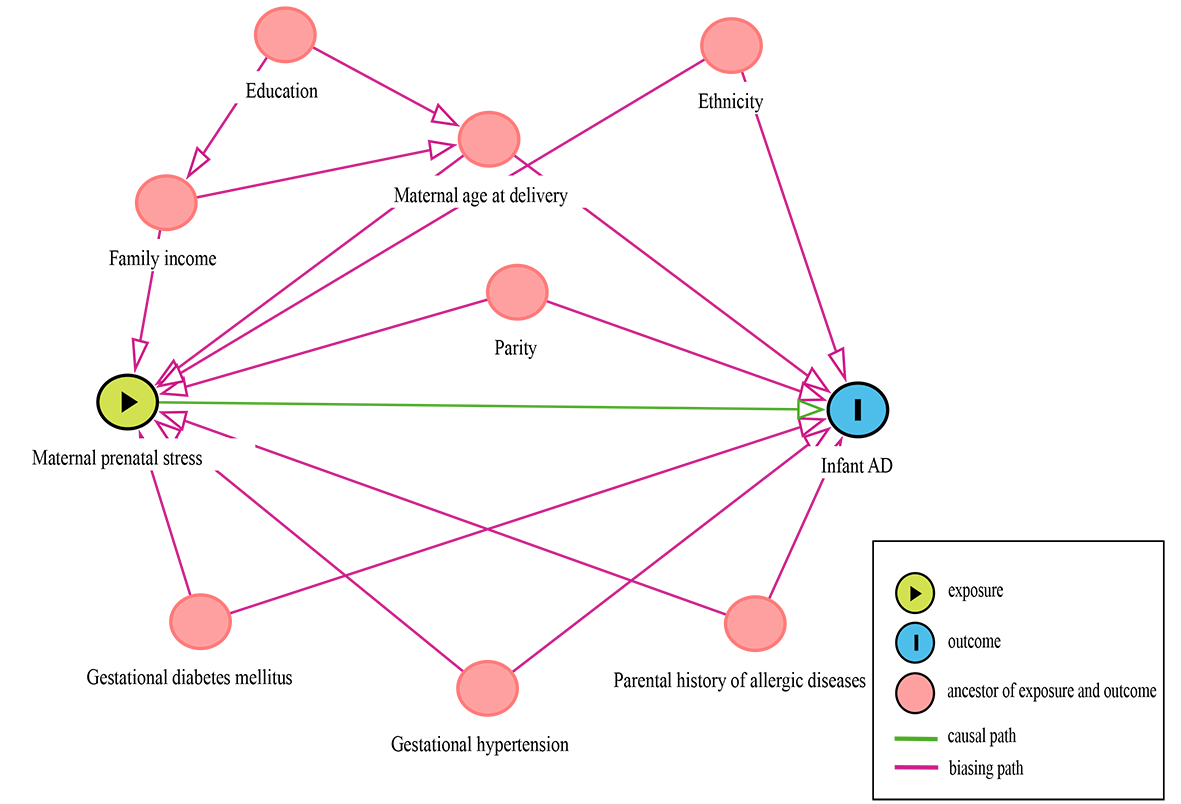

Supplement: Supplementary file 4 [file Image_1.TIF]
